# 2-Amino-5-nitro-*N*-[(*E*)-thio­phen-2-yl­methyl­idene]aniline

**DOI:** 10.1107/S1600536812037464

**Published:** 2012-09-05

**Authors:** David K. Geiger, H. Cristina Geiger, James S. Donohoe

**Affiliations:** a Department of Chemistry, State University of New York-College at Geneseo, 1 College Circle, Geneseo, NY 14454, USA

## Abstract

In the title mol­ecule, C_11_H_9_N_3_O_2_S, the thio­phene and benzene rings form a dihedral angle of 17.68 (9)°. The thio­phene S atom and the imine N atom are *syn* with respect to each other. In the crystal, N—H⋯O and N—H⋯N hydrogen bonds connect mol­ecules, forming a two-dimensional network parallel to (10-1).

## Related literature

For similar structures, see: Asiri *et al.* (2012*a* ▶,*b* ▶); Prasath *et al.* (2010 ▶). For a discussion of the use of Schiff base compounds containing thio­phene in fluorescent chemosensors, see: Chen *et al.* (2012 ▶). For a review of the biological use of 2-thio­phenes, see Kleemann *et al.* (2006 ▶). For a crystal structure from a related study on thio­phene-substituted benzimidazoles, see: Geiger *et al.* (2012 ▶).
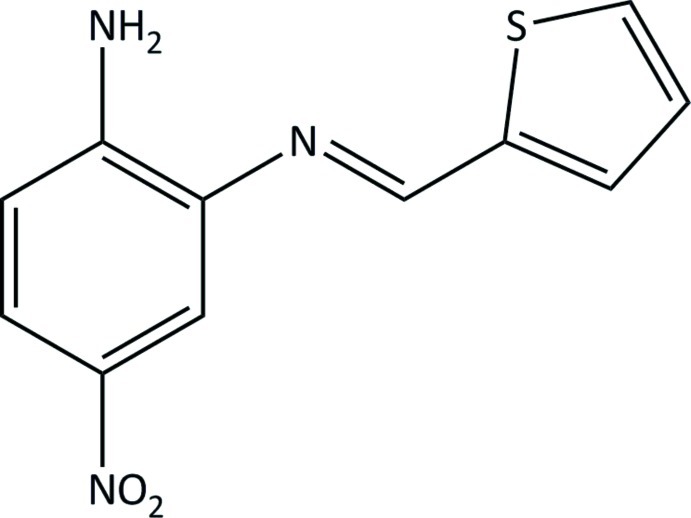


## Experimental

### 

#### Crystal data

C_11_H_9_N_3_O_2_S
*M* *_r_* = 247.27
Monoclinic, 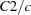

*a* = 24.335 (4) Å
*b* = 7.2084 (10) Å
*c* = 16.932 (3) Å
β = 133.396 (10)°
*V* = 2158.3 (6) Å^3^
*Z* = 8
Mo *K*α radiation
μ = 0.29 mm^−1^
*T* = 200 K
0.60 × 0.30 × 0.20 mm

#### Data collection

Bruker SMART X2S benchtop diffractometer
Absorption correction: multi-scan (*SADABS*; Sheldrick, 1996 ▶) *T*_min_ = 0.844, *T*_max_ = 0.944
6460 measured reflections
1923 independent reflections
1619 reflections with *I* > 2σ(*I*)
*R*_int_ = 0.072

#### Refinement

*R*[*F* ^2^ > 2σ(*F* ^2^)] = 0.050
*wR*(*F* ^2^) = 0.138
*S* = 1.09
1923 reflections
166 parameters
H atoms treated by a mixture of independent and constrained refinement
Δρ_max_ = 0.45 e Å^−3^
Δρ_min_ = −0.46 e Å^−3^

### 

Data collection: *APEX2* (Bruker, 2010 ▶); cell refinement: *SAINT* (Bruker, 2009 ▶); data reduction: *SAINT*; program(s) used to solve structure: *SHELXS97* (Sheldrick, 2008 ▶); program(s) used to refine structure: *SHELXL97* (Sheldrick, 2008 ▶); molecular graphics: *XSHELL* (Bruker, 2004 ▶) and *Mercury* (Macrae *et al.*, 2008 ▶); software used to prepare material for publication: *publCIF* (Westrip, 2010 ▶).

## Supplementary Material

Crystal structure: contains datablock(s) I, global. DOI: 10.1107/S1600536812037464/lh5523sup1.cif

Supplementary material file. DOI: 10.1107/S1600536812037464/lh5523Isup2.mol

Structure factors: contains datablock(s) I. DOI: 10.1107/S1600536812037464/lh5523Isup3.hkl

Supplementary material file. DOI: 10.1107/S1600536812037464/lh5523Isup4.cml

Additional supplementary materials:  crystallographic information; 3D view; checkCIF report

## Figures and Tables

**Table 1:** Hydrogen-bond geometry (Å, °)

*D*—H⋯*A*	*D*—H	H⋯*A*	*D*⋯*A*	*D*—H⋯*A*
N1—H*B*⋯O1^i^	0.81 (2)	2.25 (2)	2.991 (2)	152 (2)
N1—H*A*⋯N2^ii^	0.88 (2)	2.43 (3)	3.295 (2)	164.9 (19)

Symmetry codes: (i) 
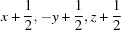
; (ii) 
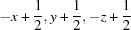
.

**Fig. 1.**
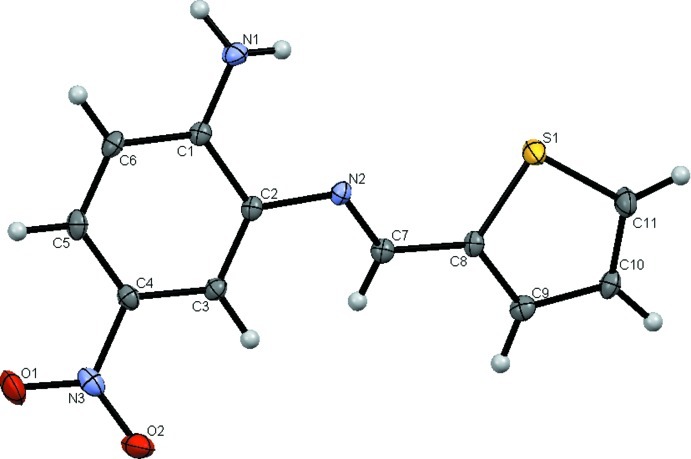
Perspective view of the title compound with displacement ellipsoids of non-hydrogen atoms drawn at the 50% probability level.

**Fig. 2.**
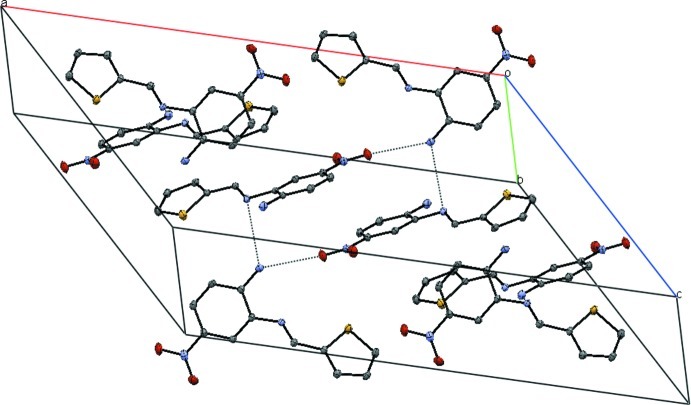
Unit cell packing diagram of the title compound displaying the donor-acceptor distances of the hydrogen-bonding network as dashed lines. H atoms are not shown and displacement ellipsoids are displayed at the 25% probability level.

## supplementary crystallographic information

### Comment

Besides their pharmacolgical importance (Kleemann *et al.*, 2006), thiophene-containing compounds are of interest because of there potential use in chemical sensors (Chen *et al.*, 2012). The title compound was isolated during our continuing studies of thiophene-substituted benzimidazoles (Geiger *et al.*, 2012).

The title compound exhibits *syn* geometry about the imine group. A perspective view of the compound is shown in Figure 1. The thiophene and benzene rings are slightly tilted with an interplanar angle of 17.58 (9)°. The imine group displays a torsional angle (C2-N2-C7-C8) of 178.5 (2)°. The plane of the nitro group is 3.6 (2)° out of the benzene ring mean plane.

A two dimensional hydrogen-bonded network (Fig. 2) emanating from the amino group and extending to a nitro oxygen atom and an imine nitrogen atom connects symmetry related molecules parallel to (101).

### Experimental

0.500 g (3.26 mmol) 4-Nitro-1,2-diaminobenzene and 1.3 ml (6.5 mmole) 2-thiophenecarboxaldehyde were stirred in 130 ml ethanol under nitrogen for 3 days. After removal of the solvent *via* rotory evaporation, the crude product was recrystallized from equal volumes of dichloromethane and diethylether. A golden solid was obtained in 70% yield. ^1^H NMR spectrum (CDCl_3_, 400 MHz, p.p.m.): 8.76 (1*H*, s), 7.99 (2*H*, m), 7.55 (2*H*, m), 7.17 (1*H*, t), 6.70 (1*H*, d), 5.00 (2*H*, bs).

Single crystals were obtained *via* vapor diffusion of hexanes into a concentrated 1-propanol solution of the title compound.

### Refinement

The amine hydrogen atoms (HA, HB) and the imine hydrogen atom (H7) were refined isotropically. All other hydrogen atoms were refined using a riding model (AFIX 43). The hydrogen atom thermal parameters were set using the approximation *U*_iso_ = 1.2*U*_eq_(C).

### Crystal data

**Table:**

C_11_H_9_N_3_O_2_S	*F*(000) = 1024
*M**_r_* = 247.27	*D*_x_ = 1.522 Mg m^−^^3^
Monoclinic, *C*2/*c*	Mo *K*α radiation, λ = 0.71073 Å
Hall symbol: -C 2yc	Cell parameters from 2950 reflections
*a* = 24.335 (4) Å	θ = 2.3–25.0°
*b* = 7.2084 (10) Å	µ = 0.29 mm^−^^1^
*c* = 16.932 (3) Å	*T* = 200 K
β = 133.396 (10)°	Plate, orange
*V* = 2158.3 (6) Å^3^	0.60 × 0.30 × 0.20 mm
*Z* = 8	

### Data collection

**Table:**

Bruker SMART X2S benchtop diffractometer	1923 independent reflections
Radiation source: XOS X-beam microfocus source	1619 reflections with *I* > 2σ(*I*)
Doubly curved silicon crystal monochromator	*R*_int_ = 0.072
ω scans	θ_max_ = 25.1°, θ_min_ = 3.1°
Absorption correction: multi-scan (*SADABS*; Sheldrick, 1996)	*h* = −25→28
*T*_min_ = 0.844, *T*_max_ = 0.944	*k* = −8→8
6460 measured reflections	*l* = −20→20

### Refinement

**Table:**

Refinement on *F*^2^	Primary atom site location: structure-invariant direct methods
Least-squares matrix: full	Secondary atom site location: difference Fourier map
*R*[*F*^2^ > 2σ(*F*^2^)] = 0.050	Hydrogen site location: inferred from neighbouring sites
*wR*(*F*^2^) = 0.138	H atoms treated by a mixture of independent and constrained refinement
*S* = 1.09	*w* = 1/[σ^2^(*F*_o_^2^) + (0.0862*P*)^2^ + 0.1606*P*] where *P* = (*F*_o_^2^ + 2*F*_c_^2^)/3
1923 reflections	(Δ/σ)_max_ < 0.001
166 parameters	Δρ_max_ = 0.45 e Å^−^^3^
0 restraints	Δρ_min_ = −0.46 e Å^−^^3^

### Special details

**Table:**

Geometry. All e.s.d.'s (except the e.s.d. in the dihedral angle between two l.s. planes) are estimated using the full covariance matrix. The cell e.s.d.'s are taken into account individually in the estimation of e.s.d.'s in distances, angles and torsion angles; correlations between e.s.d.'s in cell parameters are only used when they are defined by crystal symmetry. An approximate (isotropic) treatment of cell e.s.d.'s is used for estimating e.s.d.'s involving l.s. planes.
Refinement. Refinement of *F*^2^ against ALL reflections. The weighted *R*-factor *wR* and goodness of fit *S* are based on *F*^2^, conventional *R*-factors *R* are based on *F*, with *F* set to zero for negative *F*^2^. The threshold expression of *F*^2^ > σ(*F*^2^) is used only for calculating *R*-factors(gt) *etc*. and is not relevant to the choice of reflections for refinement. *R*-factors based on *F*^2^ are statistically about twice as large as those based on *F*, and *R*- factors based on ALL data will be even larger.

### Fractional atomic coordinates and isotropic or equivalent isotropic displacement parameters (Å^2^)

**Table:**

	*x*	*y*	*z*	*U*_iso_*/*U*_eq_	
S1	0.37474 (3)	−0.02144 (9)	0.15352 (4)	0.0390 (3)	
O1	−0.11073 (9)	0.1684 (3)	−0.18166 (15)	0.0536 (5)	
O2	−0.06193 (9)	0.0037 (3)	−0.22717 (13)	0.0485 (5)	
N1	0.23171 (10)	0.3033 (3)	0.22589 (14)	0.0317 (4)	
HA	0.2348 (12)	0.384 (3)	0.268 (2)	0.033 (6)*	
HB	0.2669 (13)	0.292 (3)	0.230 (2)	0.040 (7)*	
N2	0.22136 (9)	0.0875 (2)	0.08347 (13)	0.0246 (4)	
N3	−0.05550 (10)	0.1071 (3)	−0.16331 (15)	0.0356 (5)	
C1	0.16213 (10)	0.2554 (3)	0.13152 (15)	0.0227 (4)	
C2	0.15340 (11)	0.1461 (3)	0.05347 (15)	0.0225 (4)	
C3	0.08196 (11)	0.0985 (3)	−0.04247 (15)	0.0250 (5)	
H3	0.0758	0.0245	−0.0945	0.030*	
C4	0.01887 (11)	0.1588 (3)	−0.06301 (16)	0.0268 (5)	
C5	0.02641 (12)	0.2658 (3)	0.01241 (18)	0.0318 (5)	
H5	−0.0171	0.3059	−0.0024	0.038*	
C6	0.09727 (12)	0.3126 (3)	0.10818 (17)	0.0305 (5)	
H6	0.1025	0.3853	0.1598	0.037*	
C7	0.21931 (11)	0.0503 (3)	0.00748 (16)	0.0243 (4)	
H7	0.1755 (11)	0.058 (3)	−0.0645 (17)	0.020 (5)*	
C8	0.28411 (11)	−0.0148 (3)	0.02707 (16)	0.0242 (5)	
C9	0.28193 (11)	−0.0775 (3)	−0.05083 (16)	0.0285 (5)	
H9	0.2371	−0.0815	−0.1260	0.034*	
C10	0.35331 (12)	−0.1360 (3)	−0.00832 (17)	0.0304 (5)	
H10	0.3616	−0.1855	−0.0514	0.037*	
C11	0.40851 (13)	−0.1137 (3)	0.10053 (18)	0.0377 (6)	
H11	0.4600	−0.1455	0.1427	0.045*	

### Atomic displacement parameters (Å^2^)

**Table:**

	*U*^11^	*U*^22^	*U*^33^	*U*^12^	*U*^13^	*U*^23^
S1	0.0295 (4)	0.0638 (5)	0.0252 (4)	0.0090 (2)	0.0193 (3)	0.0003 (2)
O1	0.0246 (9)	0.0738 (13)	0.0535 (11)	0.0082 (8)	0.0235 (8)	0.0051 (9)
O2	0.0351 (10)	0.0698 (12)	0.0314 (9)	−0.0101 (8)	0.0193 (8)	−0.0121 (8)
N1	0.0313 (10)	0.0408 (11)	0.0265 (9)	−0.0039 (8)	0.0213 (9)	−0.0060 (8)
N2	0.0262 (9)	0.0252 (9)	0.0284 (8)	0.0010 (7)	0.0210 (8)	−0.0007 (7)
N3	0.0254 (10)	0.0443 (11)	0.0340 (10)	0.0012 (8)	0.0192 (8)	0.0081 (8)
C1	0.0269 (10)	0.0232 (9)	0.0238 (9)	0.0013 (8)	0.0196 (8)	0.0035 (8)
C2	0.0262 (10)	0.0225 (10)	0.0253 (10)	0.0023 (8)	0.0202 (9)	0.0041 (8)
C3	0.0280 (11)	0.0266 (11)	0.0255 (9)	−0.0004 (8)	0.0204 (9)	−0.0007 (8)
C4	0.0230 (10)	0.0311 (11)	0.0282 (10)	0.0000 (8)	0.0183 (9)	0.0031 (8)
C5	0.0315 (11)	0.0356 (11)	0.0413 (11)	0.0036 (9)	0.0301 (10)	0.0046 (10)
C6	0.0380 (12)	0.0325 (11)	0.0344 (11)	0.0023 (9)	0.0300 (10)	−0.0015 (9)
C7	0.0277 (11)	0.0238 (10)	0.0256 (10)	0.0012 (8)	0.0199 (9)	0.0037 (8)
C8	0.0269 (11)	0.0242 (10)	0.0288 (10)	0.0023 (8)	0.0220 (10)	0.0043 (8)
C9	0.0335 (11)	0.0312 (11)	0.0286 (10)	−0.0019 (9)	0.0243 (10)	0.0016 (9)
C10	0.0371 (12)	0.0322 (11)	0.0377 (11)	0.0039 (9)	0.0317 (11)	0.0020 (9)
C11	0.0304 (12)	0.0523 (14)	0.0373 (11)	0.0091 (10)	0.0259 (10)	0.0046 (10)

### Geometric parameters (Å, º)

**Table:**

S1—C11	1.713 (2)	C3—H3	0.9500
S1—C8	1.721 (2)	C4—C5	1.392 (3)
O1—N3	1.235 (2)	C5—C6	1.369 (3)
O2—N3	1.232 (2)	C5—H5	0.9500
N1—C1	1.350 (3)	C6—H6	0.9500
N1—HA	0.88 (2)	C7—C8	1.449 (3)
N1—HB	0.81 (2)	C7—H7	0.92 (2)
N2—C7	1.281 (3)	C8—C9	1.360 (3)
N2—C2	1.420 (2)	C9—C10	1.413 (3)
N3—C4	1.440 (3)	C9—H9	0.9500
C1—C6	1.400 (3)	C10—C11	1.351 (3)
C1—C2	1.424 (3)	C10—H10	0.9500
C2—C3	1.378 (3)	C11—H11	0.9500
C3—C4	1.391 (2)		
			
C11—S1—C8	91.44 (10)	C6—C5—H5	120.4
C1—N1—HA	117.8 (14)	C4—C5—H5	120.4
C1—N1—HB	118.2 (18)	C5—C6—C1	121.34 (18)
HA—N1—HB	119 (2)	C5—C6—H6	119.3
C7—N2—C2	118.08 (16)	C1—C6—H6	119.3
O2—N3—O1	122.40 (19)	N2—C7—C8	123.55 (18)
O2—N3—C4	119.38 (17)	N2—C7—H7	122.0 (12)
O1—N3—C4	118.23 (19)	C8—C7—H7	114.4 (12)
N1—C1—C6	120.86 (18)	C9—C8—C7	125.06 (19)
N1—C1—C2	120.42 (17)	C9—C8—S1	111.09 (15)
C6—C1—C2	118.72 (17)	C7—C8—S1	123.84 (15)
C3—C2—N2	124.30 (16)	C8—C9—C10	112.96 (18)
C3—C2—C1	119.68 (17)	C8—C9—H9	123.5
N2—C2—C1	115.98 (17)	C10—C9—H9	123.5
C2—C3—C4	119.93 (17)	C11—C10—C9	112.44 (18)
C2—C3—H3	120.0	C11—C10—H10	123.8
C4—C3—H3	120.0	C9—C10—H10	123.8
C3—C4—C5	121.12 (18)	C10—C11—S1	112.05 (16)
C3—C4—N3	119.41 (17)	C10—C11—H11	124.0
C5—C4—N3	119.47 (17)	S1—C11—H11	124.0
C6—C5—C4	119.21 (17)		

### Hydrogen-bond geometry (Å, º)

**Table:**

*D*—H···*A*	*D*—H	H···*A*	*D*···*A*	*D*—H···*A*
N1—H*B*···O1^i^	0.81 (2)	2.25 (2)	2.991 (2)	152 (2)
N1—H*A*···N2^ii^	0.88 (2)	2.43 (3)	3.295 (2)	164.9 (19)

Symmetry codes: (i) *x*+1/2, −*y*+1/2, *z*+1/2; (ii) −*x*+1/2, *y*+1/2, −*z*+1/2.
